# Response to Letters to the Editor Regarding ‘Role of Evogliptin for Intra‐Class Therapeutic Interchange in Patients With Type 2 Diabetes Mellitus: Subgroup Analysis From a Multi‐Center, Prospective, Observational Study (REVISE Study)’

**DOI:** 10.1002/edm2.70300

**Published:** 2026-08-27

**Authors:** Jie‐Eun Lee, Jun Hwa Hong, Min Soo Song, Jong Chul Won

**Affiliations:** ^1^ Division of Endocrinology and Metabolism, Department of Internal Medicine CHA Gangnam Medical Center, CHA University Seoul Korea; ^2^ Department of Internal Medicine, Daejeon Eulji Medical Center Eulji University Daejeon Korea; ^3^ Department of Internal Medicine Global Health Care Esoo Hospital Asan Korea; ^4^ Division of Endocrinology and Metabolism, Department of Internal Medicine Gimpo Woori Hospital Gimpo Korea

Dear Editor,

We thank Dr. Sikandar, Dr. Hashmi, and colleagues for their letters regarding our article, “Role of Evogliptin for Intra‐Class Therapeutic Interchange in Patients With Type 2 Diabetes Mellitus: Subgroup Analysis From a Multi‐Center, Prospective, Observational Study (REVISE Study)” [1]. We appreciate the opportunity to clarify the objectives of our study and discuss the interpretation of our findings.

We acknowledge the methodological issues raised, including regression to the mean, potential changes in medication adherence after treatment modification, confounding by indication, and the interpretation of subgroup analyses. These are recognized considerations in observational effectiveness research. At the same time, we believe it is important to distinguish between studies designed to establish comparative pharmacological efficacy under controlled experimental conditions and those intended to evaluate clinical effectiveness in routine practice. The REVISE study belongs to the latter category.

## 1 Study Objective and Interpretation

The principal objective of the present analysis was not to demonstrate that evogliptin is pharmacologically superior to all other DPP‐4 inhibitors. Rather, the study sought to evaluate whether patients with inadequately controlled Type 2 diabetes who underwent a clinically indicated intra‐class therapeutic interchange experienced meaningful improvements in glycemic control under routine clinical practice [2]. This represents a pragmatic clinical question frequently encountered by physicians. In everyday practice, treatment decisions are influenced not only by pharmacological considerations but also by patient preference, therapeutic inertia, tolerability, accessibility, reimbursement, and physician judgment. Consequently, the effectiveness of a therapeutic strategy reflects the combined impact of these real‐world factors rather than the isolated pharmacological properties of a single molecule. Accordingly, our findings should be interpreted as demonstrating an association between switching to evogliptin and improved glycemic control in routine clinical care, rather than establishing definitive drug‐specific superiority.

## 2 Regression to the Mean

This phenomenon is an inherent concern whenever patients are enrolled because of elevated baseline HbA1c values and should be considered when interpreting observational studies. However, HbA1c reflects average glycemic exposure over approximately 2–3 months rather than short‐term biological fluctuation. Furthermore, patients were prospectively followed for 24 weeks within a multicenter observational cohort, allowing assessment of sustained clinical outcomes rather than immediate post‐screening changes. Without a comparator group continuing the previous DPP‐4 inhibitor, the relative contributions of regression to the mean, routine follow‐up, lifestyle modification, and therapeutic interchange cannot be completely separated. Nevertheless, regression to the mean does not negate the clinical observation itself. Instead, it highlights that the magnitude of HbA1c reduction should be interpreted within the broader context of routine diabetes management, where treatment modifications are made in response to ongoing clinical needs.

## 3 Medication Adherence and the “Clinical Reset” Effect

Medication adherence is an important determinant of glycemic control, and switching therapy may enhance patient engagement, physician attention, and short‐term adherence. Because the REVISE study did not include objective adherence measurements, we cannot determine the extent to which improved adherence contributed to the observed HbA1c reduction. Importantly, however, improved adherence following treatment modification should not necessarily be viewed solely as a source of bias. In routine clinical practice, therapeutic success results from multiple interacting factors, including pharmacological activity, physician‐patient communication, treatment acceptance, and adherence [3]. The REVISE study was designed to capture the overall effectiveness associated with intra‐class therapeutic interchange rather than to isolate the contribution of each individual mechanism.

## 4 Confounding by Indication and Heterogeneity of Background Therapy

Regarding confounding by indication, treatment‐selection bias, and differences in baseline therapies: In routine clinical practice, physicians may be more inclined to switch therapies based on patient adherence, disease trajectory, and the presence of comorbidities [4]. Such heterogeneity is unavoidable in real‐world settings, where treatment decisions are individualized. To reduce measured confounding, we performed multivariable regression analyses incorporating important baseline covariates. Nevertheless, we recognize that statistical adjustment cannot eliminate residual confounding arising from unmeasured clinical variables. Future investigations utilizing propensity score methods or target‐trial emulation would further strengthen causal inference and reduce treatment‐selection bias. Rather than representing a methodological flaw unique to this study, these limitations are inherent to observational research, and they simultaneously enhance the external validity and generalizability of the findings by reflecting the complexity of everyday diabetes care.

## 5 Interpretation of Subgroup Analyses

As noted, the differences in patient numbers across the prior DPP‐4 inhibitor groups limit the precision of the estimates in smaller subgroups. Furthermore, without formal statistical interaction testing, the observed differences in HbA1c reduction among these subgroups cannot definitively prove drug‐specific pharmacological superiority. Therefore, the subgroup results should be interpreted with caution. They serve as exploratory, hypothesis‐generating findings rather than conclusive evidence of differential treatment responses among the prior DPP‐4 inhibitors.

## 6 Conclusion

We acknowledge the authors of both letters for highlighting the distinction between observational effectiveness and causal comparative efficacy. Regression to the mean, adherence‐related effects, confounding by indication, and the exploratory nature of the subgroup analyses limit causal attribution in our study. However, these considerations should be viewed within the context of the study's pragmatic objective. The REVISE study was designed to evaluate how intra‐class therapeutic interchange performs under the complexity of routine clinical practice. Taken together, the REVISE sub‐analysis contributes pragmatic evidence supporting intra‐class therapeutic interchange to evogliptin as a feasible, safe, and clinically relevant treatment strategy for selected patients, generating hypotheses that warrant further investigation in appropriately designed randomized comparative studies.

## Author Contributions

**Jun Hwa Hong:** conceptualization, writing – original draft. **Min Soo Song:** supervision, conceptualization. **Jie‐Eun Lee:** conceptualization, writing – original draft. **Jong Chul Won:** conceptualization, supervision.

## Conflicts of Interest

The authors declare no conflicts of interest.

## Linked Articles

This article is linked to Sikandar papers. To view this article, visit https://doi.org/10.1002/edm2.70283.

## Data Availability

The data that support the findings of this study are available from the corresponding author upon reasonable request.
